# Beliefs, attitudes and experiences of virtual overdose monitoring services from the perspectives of people who use substances in Canada: a qualitative study

**DOI:** 10.1186/s12954-023-00807-9

**Published:** 2023-06-24

**Authors:** Tyler Marshall, Dylan Viste, Stephanie Jones, Julia Kim, Amanda Lee, Farah Jafri, Oona Krieg, S. Monty Ghosh

**Affiliations:** 1 Department of Medicine, Cumming School of Medicine, University of Calgary, Calgary, AB Canada; 2 Three Hive Consulting, Vancouver, BC Canada; 3 Department of Medicine, Faculty of Medicine & Dentistry, University of Alberta, Edmonton, AB Canada; 4 Department of Internal Medicine, Faculty of Medicine & Dentistry, University of Alberta, Edmonton, AB Canada; 5 BRAVE CO-OP, Vancouver, BC Canada

**Keywords:** E-health, Harm reduction, People who use substances, Addictions, Canada, Virtual overdose monitoring services

## Abstract

**Background:**

Solitary use of substances is a risk factor for substance use-related mortality. Novel e-health harm reduction interventions such as virtual overdose monitoring services (VOMS) have emerged in North America to improve access to emergency overdose support for people who use substances (PWUS). To date, little research has been published, and the perspectives of PWUS are needed to inform evaluation and policy efforts.

**Objective:**

To explore the beliefs, values and perceptions of PWUS around using and accessing VOMS in Canada.

**Methods:**

A qualitative study following grounded theory methodology was conducted. Using existing peer networks, purposive and snowball sampling was conducted to recruit PWUS (≥ 18 years) with previous experience with VOMS. Thematic analysis was used to analyze twenty-three interviews. Several methods were employed to enhance rigor, such as independent data coding and triangulation.

**Results:**

Twenty-three one-on-one telephone interviews of PWUS with previous experience with VOMS were completed and analyzed. The following themes emerged: (1) feelings of optimism around VOMS to save lives; (2) privacy/confidentiality was highly valued due to stigma and fear of arrest; (3) concerns with reliable cell phones negatively impacting VOMS uptake; (4) concerns around emergency response times, specifically in rural/remote communities; (5) desire for trusting relationships with VOMS operators; (6) importance of mental health supports and referrals to psychosocial services; and (7) possible limited uptake due to low public awareness of VOMS.

**Conclusion:**

This qualitative study provided novel insights about the VOMS from the perspectives of PWUS. PWUS generally felt optimistic about the potential of VOMS as a suitable harm reduction intervention, but several potential barriers around accessing VOMS were identified that may limit uptake. Future research is warranted.

**Supplementary Information:**

The online version contains supplementary material available at 10.1186/s12954-023-00807-9.

## Highlights

- Virtual overdose monitoring services (VOMS) are novel harm reduction services that function by linking a person who uses drugs with timely emergency care during a suspected overdose via electronic communication.
- Twenty-three people who use substances were interviewed exploring their perceptions and experiences with VOMS.
- This qualitative study found that people who use substances (PWUS) positively perceived VOMS as an acceptable harm reduction intervention, particularly among PWUS alone.
- Low public awareness and limited access to reliable technology may limit the rapid uptake of VOMS.
- More research is needed to explore the feasibility of VOMS in rural geographic areas and in minority populations.

**Supplementary Information:**

The online version contains supplementary material available at 10.1186/s12954-023-00807-9.

## Introduction

In recent years, substance use-related mortality rates have risen to epidemic levels across North America. In 2020, the unadjusted incidence rates of substance use-related mortality were 76.9 per 1,000,000 people [10, 31] in the USA and 171.1 per 1,000,000 people in Canada [24, 27]. These marked increased rates of substance-related mortality are largely related to recent widespread changes to the unregulated drug supply, with fentanyl and its analogues (e.g., carfentanil) becoming the predominant opioid of non-medical use, including increased reports of fentanyl contamination in other recreational drugs such as cocaine and methamphetamine [20]. In addition to an increasingly toxic drug supply, solitary substance use, specifically, the use of drugs without another individual present to administer an emergency response (e.g., CPR, naloxone, is also strongly associated with the recent increased substance-related mortality rates, particularly fentanyl-related deaths [18]. In 2018, studies found that 69% of overdose deaths in the province of British Columbia and 56% of opioid overdose deaths in the USA appeared to h occurred during solitary use [15, 17].

Evidence from systematic reviews suggests that supervised consumption services are effective at preventing opioid use-related mortality without increasing public disorder [12, 23], but implementation (i.e., socioeconomic, legal and political) issues may have limited widespread uptake of these services [19]. However, for some PWUS, attending SCS may not always be ideal due to concerns about transportation/convenience, privacy [30], harassment by police or arrest [2]. For instance, the authors of one study reported that 53% of PWUS believed using substances at home was more convenient [30].

In recent years, research around virtual and telehealth for addiction medicine has increased across North America, largely due to necessities associated with the COVID-19 pandemic [6], while research on virtual harm reduction services remains largely unexplored [14]. As a result, little is known about the role of virtual harm reduction measures as a method to combat the drug poisoning crisis. Donnell et al. [5] conducted a scoping review of Canadian and Australian studies and only found three studies involving “digital technologies for overdose monitoring and prevention.” The authors suggested that more qualitative studies are needed to inform these services' policy, practice and delivery [5].

Virtual overdose monitoring services (VOMS) are based on the idea of “drug spotting” [21, 22] is a long-standing and informal practice among PWUS, which involves the monitoring of an individual who is using substances and, in the event of an overdose/poisoning, facilitating an emergency response (e.g., calling emergency services, performing CPR, administering naloxone). While VOMS employ harm reduction-based and nonjudgmental approaches to care, provision of additional services such as substance use education, mental health support and referrals to health and social services are commonplace. Although no formal name or definition exists, we define VOMS as public health services that use either telephones or digital app-based technology to monitor and facilitate emergency support to individuals who use substances alone and are at risk of a drug poisoning event.

As of 2023, various modalities for VOMS currently exist across North America. In 2019, the first virtual overdose monitoring service (e.g., VOMS) was launched in the USA, Never Use Alone (NUA), to prevent overdose mortality among people using drugs alone. In 2020, two virtual overdose monitoring services (VOMS) were launched in Canada (National Overdose Response Service (NORS), The Brave App) to provide reliable, continuous (i.e., 24 h a day) harm reduction support and substance use monitoring for people who use substances alone and for those who may be unable to access in-person supervised consumption services.

**Fig. 1:**
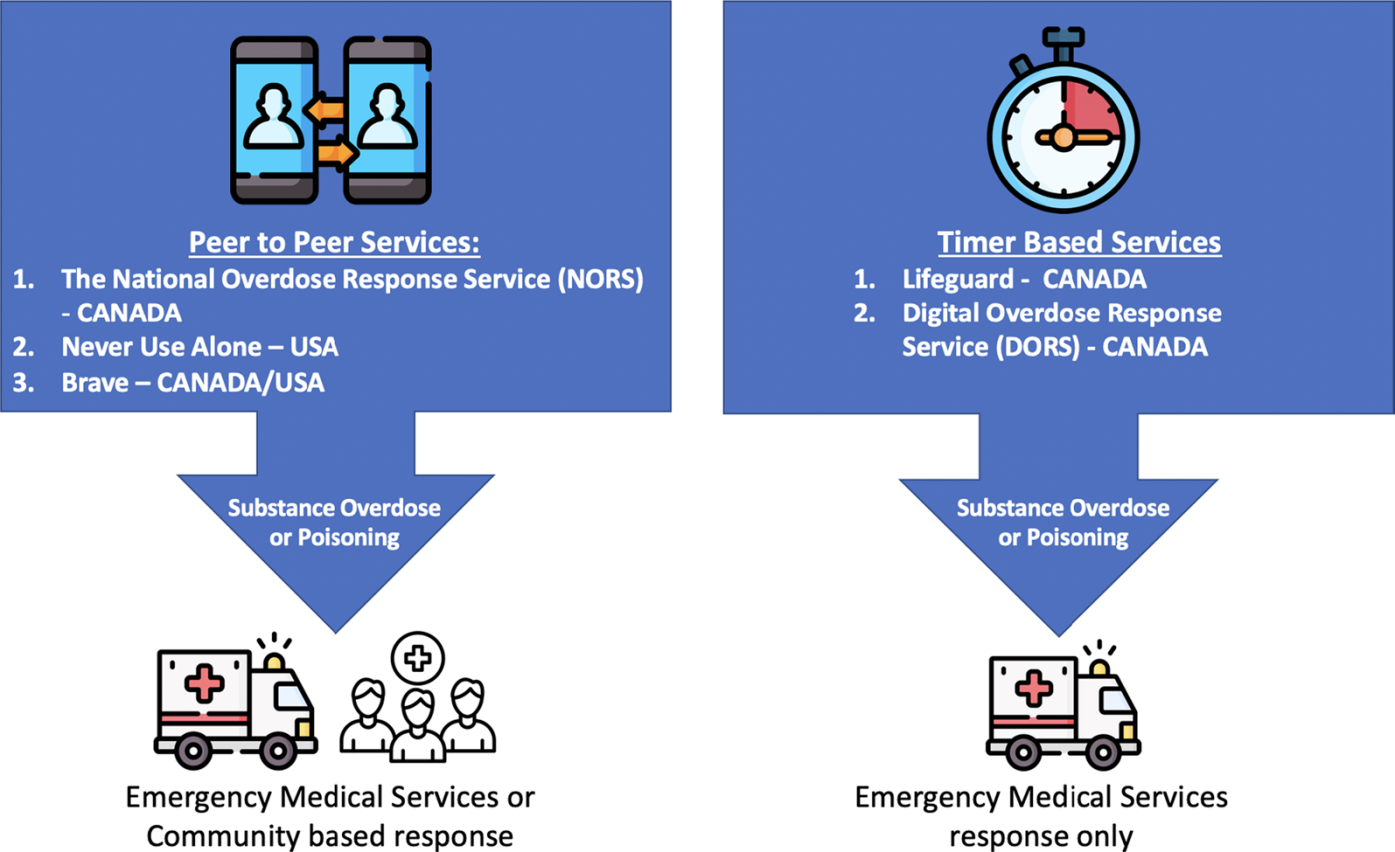
Diagram of peer-to-peer and timer-based VOMS currently operational in North America

NORS is a peer-lead (i.e., operators have lived/living experience with substance use) telephone hotline that operates Canada-wide. NORS operators can initiate a personally tailored, pre-made emergency response plan during an adverse event [14]. Other digital VOMS, such as the Digital Overdose Response System (DORS), and Lifeguard employ an automated digital timer that must be reset periodically (e.g., every 60 s) by the person using substances [14]. An emergency response plan will be activated if the caller does not manually reset the timer (e.g., due to a decrease or loss of consciousness) [14]. Figure 1 shows an example of the various VOMS active across North America.

Little is known about VOMS from the perspective of people who use substances. To ensure ongoing policies and practices are evidence-informed and person-centered, initial qualitative studies are needed from the perspectives of PWUS and diverse stakeholders to establish an evidence base of sufficient depth, breadth and quality. The objective of this study was to explore the perceptions and beliefs around VOMS from the perspectives of PWUS in Canada. This research will be used to develop hypotheses that will inform future research and service design.

## Methods

**Fig. 2:**
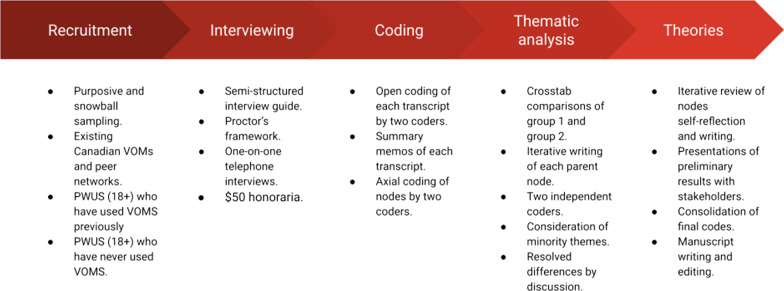
Overview of the methodology

A qualitative study was conducted that explored the perceptions and beliefs around VOMS from the perspectives of PWUS in Canada. Between February and March 2022, one-on-one telephone interviews were conducted with PWUS who have experience with VOMS (i.e., either as a client, volunteer or peer operator) over 18 years of age across Canada. It should be noted that many of the peer operators also used the VOMS regularly as a client when not on shift. Grounded theory methodology was used to guide the methods and analysis. Grounded theory was the most appropriate methodology for addressing our research question as it is commonly used to develop rich hypotheses and theories around complex phenomena involving the evaluation of public health programs [11, 16, 33]. The protocol for this study was not preregistered but may be available upon reasonable request. This study was approved by the University of Calgary Conjoint Health Research Ethics Board (REB21-1655). Figure 2 displays an overview of the methodology.

### Description of the research team

This study employed a multidisciplinary research team consisting of a postdoctoral research fellow (TM) who has previous doctoral-level training and experience in conducting qualitative interviews and analyses; an addiction medicine physician, a harm reduction researcher and VOMS co-founder (MG) who has masters-level training with previous experience conducting qualitative studies; a research assistant (DV) who has bachelors-level training, (SJ) a masters-level consultant data collector who has previous experience with conducting and analyzing qualitative research, two resident physicians (FJ and AJ), a medical student (JK) and a VOMS co-founder and peer (OK).

### Sampling strategy

Operators of two VOMS (NORS, the Brave app) purposively recruited clients and snowball sampled existing peer networks to identify potentially eligible and interested individuals engaging with VOMS. Individuals who have used a VOMS previously (i.e., as a client) in addition to VOMS operators as well as PWUS who have never used VOMS were recruited. A recruitment package was developed in collaboration with VOMS administrators, which contained a telephone recruitment script, contact information, a verbal consent form (completed by the interviewer before the interview) and an information letter. After the interview, participants were asked to recommend other potentially eligible individuals.

### Participant eligibility criteria

The following eligibility criteria were established a priori:

#### Inclusion criteria

- Canadian residents ≥ 18 years of age at the time of consent;
- Reported active use of unregulated substances (within the last seven days);
- Able to communicate effectively in English and provide informed verbal consent;
- *PWUS* who worked in harm reduction or for VOMS were eligible.

#### Rationale

We prioritized individuals who had relevant experience using VOMS (e.g., clients, volunteers, peer operators), as we believed these groups would be the most likely to have sufficient experience with VOMS to provide credible insights about the strengths and limitations of using and accessing these services, including knowledge of potential barriers for accessing the services from either their own lived experiences or contact with clients via peer networks.* We did have some PWUS who did not use VOMS, to also gain insight on why or why they would not use these services even though they knew about VOMS from our snowball sampling methodology.*

### Development of the interview guide

A semi-structured interview guide (Additional file 1) was developed with the research team, VOMS operators and two PWUS. The questions were framed in the context of Proctor’s framework, an implementation science framework for evaluating health programs [13]. This framework was used to ensure the findings would apply to the evaluation and be informative for quality improvement purposes of VOMS.

### Interview process

Participants were required to provide informed verbal consent prior to participation in the study. Sociodemographic data were collected via verbal questionnaire post-interview. Telephone interviews (ranging approximately 15 to 70 min in duration) were conducted by a trained data collector (SJ), and reflective field notes were collected by hand. Participants were free to decline questions or discontinue the interview session at any time without penalty. Mental health crisis support information was provided to participants should they feel unwell or request it during the study. The interviews were audio recorded using the *Tapeacall* app and then transcribed verbatim using a third-party transcription service, and the data were cleaned (i.e., identifiers removed) manually. Participants received a $50 prepaid gift card after participating in the study.

### Analysis

The data were analyzed using thematic analysis based on grounded theory methodology [4]. We chose this approach to promote methodological coherence aligning with the research objective [3, 8]. Two independent authors coded each transcript. Five authors (TM, DV, JJ, FJ and AM) participated in coding the transcripts using NVIVO 12.7 [25]. Consistent with grounded theory methodology, inductive and deductive methods were applied during the analysis including open, axial and selective coding methods [29]. First, open coding methods (i.e., line-by-line coding) were used to create a codebook based on initial concepts obtained from the data. Next, axial coding was performed to develop emerging themes, and nodes were aggregated into parent and child nodes. Each node was given an operational definition once saturation was agreed upon by the coders. Selective coding (examination of the data) was initially written by two coders, then again by the larger research team, and during peer-review to ensure themes adhered closely to the participant data. The coders wrote summary memos after each transcript detailing the participants’ primary viewpoints and noting any disagreements. Any discrepancies among the coders were resolved by group discussion. The coders were trained by the first author (TM), who has doctoral-level training and experience in qualitative methodology.

#### Verification strategies

Methodological rigor was maintained using several approaches. Triangulation was performed by comparing themes according to people who previously used VOMS (group 1), to PWUS who have never used VOMS (group 2). Group 1 was defined as PWUS who previously used VOMS as a client at least once. Group 2 was defined as PWUS who have never used a VOMS. Member checking involved several oral presentations of the preliminary evidence to VOMS operators identified via NORS and PWUS who were not research participants before submission of the manuscript for publication, psychiatrists (e.g., at grand rounds) and addiction specialists (e.g., at an addiction conference).

## Main findings

Twenty-three PWUS (57% men) completed the study and were analyzed. They represented individuals from across Canada and from 7/10 different provinces. Twenty-three PWUS completed interviews as two participants were excluded from the analysis. (One did not meet eligibility criteria, and the other could not adequately contribute due to confidentiality concerns.) Forty-eight percent of the sample had previous experience using substances under supervision from a VOMS (i.e., as a client, volunteer or peer operator). Table 1 displays the sociodemographic data of the PWUS who completed the study.

**Table 1:** Sociodemographic data

Variable	Total (*n* = 23)
Age, mean years (SD)	38.3 (12.0)
*Gender n (%)*	
Man	13 (56.5)
Woman	9 (39.1)
Non-binary	1 (4.3)
*Ethnicity n (%)*	
Persons of color (non-indigenous)	2 (8.7)
Indigenous	5 (21.7)
*Province of residence n (%)*	
Alberta	8 (34.8)
Nova Scotia	1 (4.3)
Ontario	13 (56.5)
Quebec	1 (4.3)
Urban residence *n* (%)	20 (87.0)
Used VOMS previously *n* (%)	11 (4**7**.8)

**Fig. 3:**
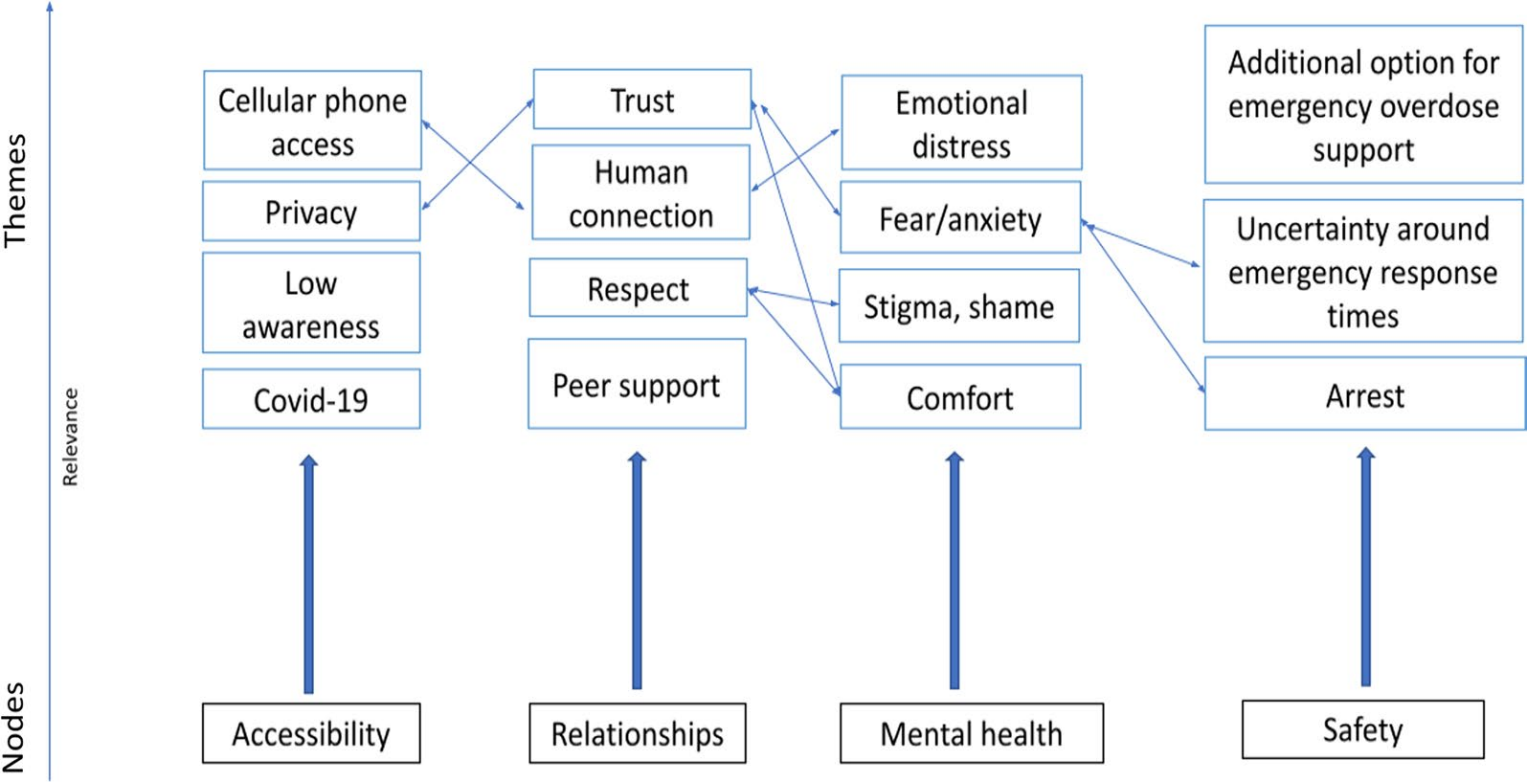
Concept map of emergent themes

The thematic analysis results describe several themes that emerged from the study population. Figure 3 provides a concept map of some of the key themes that emerged from the thematic analysis, while Table 2 provides an overview of the nodal structure of these key themes. Quotes were selected illustrating these themes with relevant demographic data. Previously, People who used VOMS generally expressed more positive attitudes around VOMS's effectiveness than those who did not; however, only some major differences in themes between the groups were identified, and the results are reported collectively. Supplementary quotes can be found for each theme in Additional file 1.

**Table 2:** Coding structure

Parent nodes (*n* participants)	Node description	Child nodes (*n* participants)
Accessibility (*n* = 23)	Any discussion about accessing or using VOMS	Access to technology and/or cellphones (*n* = 23)
		Applicable population (*n *= 22)
		Applicable setting (*n* = 17)
		Covid-19 (*n* = 17)
Awareness (*n* = 23)	Comments about previous knowledge, awareness or advertising/promotion of VOMS	Advertisement (*n* = 23)
		Previous knowledge about VOMS (*n* = 16)
Relationships (*n* = 23)	Any discussion about feelings, desires for relationships and interactions with other individuals in the context of substance use or using VOMS	Privacy/confidentiality (*n* = 23)
		Human connection (*n* = 21)
		Trust/respect (*n* = 19)
		Community (*n* = 12)
		Nonjudgmental (*n* = 11)
Potential health benefits (*n* = 22)	Any comments about possible health benefits from using VOMS	Referrals to health and social services (*n* = 15)
		Saving lives (*n* = 9)
Mental health (*n* = 22)	Any discussion about mental health or individual emotions in the context of using or accessing VOMS	Emotional distress (*n* = 18)
		Comfort (*n* = 17)
		Fear and anxiety (*n* = 16)
		Stigma, shame, guilt (*n* = 16)
Perceptions of safety concerns (*n* = 23)	Any discussion about perceptions of safety concerns or hesitations when using VOMS	Emergency response times (*n *= 20)
		Criminalization (*n *= 13)

### Theme 1

Optimistic beliefs around VOMS as an acceptable harm reduction intervention.

PWUS were generally optimistic that VOMS might be useful as an appropriate harm reduction intervention for reducing overdose deaths. They acknowledged that “spotting” already exists in the community and that VOMS is an extension of this practice in a more organized fashion.

“You know my main spotter … spots me from Seattle. And we've never had like a bad event. [Name] does a lot of these….But yeah, like I try to always have someone on the other end. Even if it's for like crack cocaine, or things like that. Because you never know.” (G2P01, 32, Man)

Some individuals provided accounts of how VOMS was already working for them and how VOMS facilitated an emergency response for them, and their overdose was reversed.

“This [telephone] line can save so many people’s lives. It saved my life.” (G1P09, 20, Woman)

### Theme 2

Feelings of privacy/confidentiality and convenience may be essential for deciding to use VOMS.

Using VOMS privately and anonymously was considered essential for deciding to use VOMS. Many PWUS reported having histories of trauma (i.e., arrest, abuse) and some distrust government-funded health services and were hesitant to provide any identifiable information. Participants suggested that requirements for providing personal health information to use VOMS might pose a barrier to using VOMS for many individuals. However, a few participants acknowledged that some personal information (e.g., address/location) is required to facilitate successful emergency responses.

“I mean had you had to give them all your information and that I think it would be a little bit more of a deterrent. Yeah, so like if you had to give them your information and, you know, it wasn’t anonymous I think a lot of people wouldn’t use it. I don’t think I would have, no.” (G1P07, 32, Woman)

Although this did not reach saturation, other variables such as an individual’s geographic distance from SCSs, weather, SCS wait times and convenience of using substances in one’s home/residence may also play a role in VOMS utilization.

“.. at certain times of the year, and I feel like it must have been winter as well, at certain times of the year SCS gets very busy, especially in Edmonton… even in Vancouver… sometimes you'd be waiting for 45 minutes to get into an SCS and it's not exactly convenient to wait 45 minutes when … you’ve just gotten something and you're trying to be safe...” (G1P12, 29, Man)

### Theme 3

Unreliable access to cellular phones or cellular service may negatively impact uptake of VOMS.

Participants generally believed that technological barriers may hinder VOMS's widespread uptake and success in reducing overdose deaths on a large scale (i.e., population level). For instance, participants commonly believed that many PWUS may need more reliable access to a cellular/mobile phone or cellular service, especially in rural or remote geographic areas, for VOMS to be viewed as a reliable service.

“… lots of people don’t have phones to access it. It’s very easy to access if you have a phone, but if you don’t it’s hard, [laughs] so, but it’s just a hit or miss, I guess.” (G1P09, 20, Woman)

### Theme 4

Concerns about the reliability of emergency response times as potential barriers to uptake

Most PWUS believed the intervention would increase safety compared to using substances alone, but some individuals were worried that emergency response times might be unpredictable or unreliable. Most participants agreed that this issue might be more of a concern for people living in rural or remote geographic areas. People who had never used VOMS previously had a more pessimistic view of the safety around VOMS (e.g., technical issues, delayed response times, privacy concerns) but generally felt using a VOMS would be safer than using it alone.

“I’m in rural Nova Scotia. I don’t think 911 would even make it here in time to be honest. So, I think there needs to be like a better like community plan in place. Where you have maybe like two community workers that are driving around giving out supplies.” (G2P01, 32, Man)

### Theme 5

Fear and anxiety of legal consequences as barriers for uptake

Fear and anxiety about stigma, judgment and legal consequences (including arrest or loss of child custody) were commonly reported among the participants, particularly those who had never previously used virtual services. Some respondents feared police presence in the event of a suspected overdose due to the fear of criminalization and suggested that “it would be great if I was 100% guaranteed that the police wouldn’t show up” (G2P07, 35, Woman). Respondents also highlighted the need to “understand [their] rights better should police attend” (G2P02, 40, Non-binary). The data strongly suggested that concerns about legal consequences were a primary psychological barrier to using a VOMS for the first time.

“I think if it was now, like I have a daughter, I would be too afraid that I would lose my kid sort of thing, so that would be the scariest. Or get arrested, find out like a warrant, you know, if the paramedics show up do the police come. You know what I mean?” (G2P07, 35, Woman)

### Theme 6

Trusting and nonjudgmental peer-to-peer relationships are commonly desired between service providers and VOMS clients.

Forming trusting relationships and human connections was important for the participants, particularly among PWUS, who had experience with telephone-based VOMS (e.g., NORS) that employ human operators. Both PWUS who have used VOMS (group 1) and have not used VOMS (group 2) agreed that a friendly, caring, informal relationship between clients and operators was essential for establishing rapport and that being treated with dignity and respect helped alleviate feelings of nervousness when using VOMS for the first time. Some respondents attributed feelings of anxiety around using substances in the presence of the unknown operator for the first time, but this may have been mitigated due to the nonjudgmental attitudes expressed by the operators.

“I was scared to call the line, because I know there’s a lot of judgement in the addiction community […] but I ended up calling anyway […] it surprised me how great they were […] Almost every person I talked to there, were so understanding and so nice […] some of the people that were on the line I got pretty close to […] I got to build actually some relationships with a few of them, which was great.” (G1P11, 39, Man)

While the desire to form relationships with operators is only applicable to peer-to-peer-based services, a minority of PWUS did prefer VOMS that used digital timers as they held more skeptical views of government-funded interventions and preferred the further anonymity that a digital timing-based service afforded with no person-to-person/peer-to-peer interaction. Additionally, others preferred using substances alone, without peer operators or automatic timing apps, citing concerns about confidentiality that VOMS services could lose.

### Theme 7

Importance of providing mental health support, peer support and referrals to psychosocial services

Participants suggested that PWUS might experience complex mental health needs such as anxiety, depression, loneliness, isolation and psychosis or schizophrenia. Participants suggested that people who use VOMS who may be worried about consequences such as fear of arrest could leverage the provision of mental health and peer support. Operators can provide adequate information and take a trauma-informed and individualized approach which might reduce some discomfort.

“Yes, well I was definitely worried about the police being called just about anything. And, I don’t know, with me I do have a long history of trauma and stuff. So there’s some people that I just – I don’t know – I prefer to speak to like females. And they were really great with that. If I wanted to speak to a female, I just called back and they set me up with a female.” (G1P09, 20, Woman)

Participants appreciated that VOMS were not only harm reduction interventions but might be entry points for obtaining timely additional health and social services as requested.

“So I have called for resources a few times, yes. They’re really great with connecting – they have a huge resource list across Canada and there’s been a few times in different cities where my in-laws live where we asked for help and resources out there and they can always point us in the right direction.” (G1P05, 38, Woman)

### Theme 8

Low public knowledge/awareness about VOMS among many PWUS may also contribute to limited uptake

Participants, particularly those who have worked with VOMS previously, believed that uptake for using VOMS could be higher. Participants commonly believed that, despite available information, more awareness of VOMS is needed to improve uptake. Participants believed it would be important to know what VOMS offer, who was eligible to use them, how they might be accessed, how long the emergency response times are and what would be done to protect their privacy. Most participants reported learning about VOMS via word of mouth rather than via online or social media.

“No, I think that there's a lot of information, I just wish that there was more callers calling in.” (G1P01, 41, Woman)

COVID-19 may have played a minor role in the uptake of SCS and VOMS as many SCS stayed open, but some SCS had reduced capacity, and some individuals had to quarantine while using substances.

“But for me personally, especially because the SCSs are so meticulous about cleanliness and, yeah, I think it just ended up that I just didn't use an SCS in the beginning of the pandemic when things fully locked down. But I did find out soon after that I think one of them was open still anyways, so yeah, that wasn't really a factor for me personally, but I could it see being a factor for other people maybe immunocompromised or, yeah, something like that.” (G1P12, 29, Man)

## Discussion

To our knowledge, this is the first study to explore the beliefs and attitudes around VOMS from the perspective of PWUS. In this study, we found that the PWUS generally held favorable views about VOMS as an acceptable harm reduction intervention, specifically for PWUS with reliable access to a cell phone. The participants identified several themes around the values of PWUS and possible strengths of VOMS. PWUS were generally optimistic that VOMS have the potential be an effective public health intervention capable of mitigating the risk of overdose deaths for those who use these services. They highlighted that “drug spotting” is already being done informally in substance-using communities and that VOMS help formalize this practice. People who had never used VOMS previously held more skeptical views regarding the reliability of VOMS to facilitate timely emergency response times and may be particularly unpredictable/unreliable for people living in rural/remote geographic areas. Despite these potential risks, both groups supported VOMS and thought using substances with support from these interventions may be safer than using substances alone.

Many PWUS value forming trusting relationships with peer operators. This was likely necessary due to concerns around potential privacy breaches and fear of legal consequences. Notably, most VOMS that operate like a phone hotline (e.g., NORS, NUA, the Brave app) employ PWUS to provide support, which participants considered important to feeling socially and mentally provided for since operators could relate to their clients. This finding aligns with other studies demonstrating that peer-to-peer support is a powerful tool in harm reduction and substance management, enhancing many harm reduction services [1]. A few individuals preferred to use fully automated VOMS (e.g., digital timers), which do not involve talking to another individual. This highlights the need for hotline and automatic timer VOMS options for individuals depending on their preferences and comfort levels.

Concerns around unreliable telephone access were pervasive among PWUS, and there were concerns for PWUS who live in remote or rural geographic areas. This finding is consistent with previous literature that shows that approximately 45% of PWUS have access to cell phones, and the author suggests that “technological applications may not be suitable for clients with transient lifestyles, no permanent home, and lack of consistent access to a mobile device” [30]. If future research indicates that VOMS are both cost-effective and life-preserving or life-improving, this may warrant the provision of the necessary technology to PWUS in Canada, as well as determining methods to improve access to technology in rural and remote communities.

Fear of legal consequences and stigma posed the largest psychological barriers for accessing VOMS among those with access to cell/mobile phones. This was a novel finding in the context of virtual/digital harm reduction care but is consistent with the literature around accessing harm reduction more broadly. The extant literature found that stigma and structural violence was a major barrier to accessing harm reduction among women [26]. Similar to other research, even though the *Good Samaritan Drug Overdose Act*, which protects against legal prosecution (with some exceptions) is in effect in Canada [9, 32], individuals still were concerned that using VOMS may lead to a variety of legal consequences. For instance, a few participants highlighted concerns about the limitations of the act. Within a Canadian context, the act, unfortunately, does not protect against child apprehension from parents who use substances. Additionally, individuals can still be apprehended by police if they have outstanding arrest warrants, violate probation or parole or are found trafficking substances. Lastly, the act does not prevent individuals from having their substance paraphernalia and substances in their possession from being confiscated, which places additional limitations and may increase hesitancy to use these services. Despite this, hesitation and anxiety about using the service was often reduced by interacting with the peer operators, demonstrating the ability of peer operators to help overcome these barriers, and further support uptake onto VOMS.

Consistent with related harm reduction research, we found that people who use VOMS may commonly experience complex psychosocial needs such as anxiety, depression, loneliness/isolation and sometimes psychosis [14]. Emotional distress among callers was commonly reported during interactions at telephone-based VOMS. While participants valued privacy and confidentiality, peer relationships emerged to facilitate trust and comfort for many callers. VOMS telephone operators often have lived or living experience with substance use, overdose or addiction, which appears to be a strength of peer-to-peer VOMS among the participants. We found that peer-to-peer therapeutic relationships may ease distress, provide comfort and promote client trust. As a result, we hypothesize that peer operators may be able to successfully provide additional information and connect clients to additional health and social services due to this increased therapeutic alliance. A minority of individuals noted they may not feel comfortable talking to someone they do not know during their substance use and would prefer fully private and digital VOMS such as Lifeguard, or the Digital Overdose Response System (DORS) in Alberta employs a timer-based system and no human interaction on the phone. Several individuals suggested that strong preferences for privacy/confidentiality may be a direct result of past trauma, and fear of arrest, which were also linked with past experiences of shame, judgment and guilt. These negative past experiences were also connected with reduced accessibility and hesitations for using VOMS and other harm reduction services.

The resulting themes were found to be largely interconnected. For instance, fear of legal consequences was related to emotional distress and mental health, but also related to desires for privacy and skepticism for using VOMS. These findings were consistent with previous research, which also found that stigma/shame, privacy, mental health conditions were associated with poor engagement with existing in-person harm reduction services and that these factors might contribute to solitary drug use [7]. All VOMS were seen as an entry point for PWUS to access additional mental health/social services, but low general awareness in combination with concerns around technology access, fear of arrest or child apprehensions and emergency response times may limit the effectiveness and reach of VOMS as currently delivered.

Additionally, this study builds upon previous research, provides plausible explanations for low utilization rates for VOMS and displays insights on how utilization rates might be improved [14]. Previous research suggests that approximately 350 individuals used one VOMS (NORS) during 2020–2021. Although no deaths were recorded, utilization rates remain low in comparison with SCS, and less than 10% of the callers resided in rural geographic areas [14]. Concerns about emergency response times, distrust of public health services due to fear of stigma, shame and privacy concerns all may play a role in the relatively low uptake of nationwide harm reduction services [7].

## Strengths and limitations

We note several strengths of this study. First, this study collected rich data from an adequate sample size. We included various participants from various backgrounds, genders and geographic locations across Canada. Two authors independently coded each transcript using open and axial coding methods. We developed several novel and key hypotheses that can be used to inform service design, recruitment and expansion efforts, funding and policy.

Several limitations of the study are noted. First, a broad range of interview questions led to a wide range of responses which posed challenges for themes to hit saturation. People who had telephones only could participate in the service; therefore, the results may only be generalizable to PWUS who have access to telephones. Nearly half of the sample had used VOMS previously, and several others had previous experience or were employed by VOMS. This may have led to sampling bias and impacted the interpretation of the results. However, this was unavoidable as recruiting people with active substance use disorders in research studies can be challenging. Several questions involved participant recall which may result in recall bias; however, this was an unavoidable limitation, and we do not believe this significantly impacted the results. While participants often discussed rural concerns, only some people living in rural areas participated in the study; thus, conclusions about the values of PWUS and the potential acceptability or feasibility of VOMS in rural communities remain limited and will need future evaluation.

## Conclusions

VOMS may be useful harm reduction interventions providing an adjunctive option for substance use monitoring and emergency response for PWUS alone and individuals who may experience barriers to accessing in-person SCS (e.g., due to quarantine, transportation, stigma, disability). Our data, in the context with the best available evidence, suggests VOMS may be most applicable for PWUS who have reliable access to technology, use substances alone and cannot access an in-person SCS. A novel and important finding also included that VOMS may also be beneficial at providing peer support and facilitating referrals to health and social services. However, due to potential limitations around emergency response times and lack of in-person monitoring, we suggest using in-person SCS for providing overdose monitoring and supervision when possible. Since most substance-related overdoses occur during solitary substance use and away from an SCS, VOMS may be an important public health intervention for a population at high risk of mortality. It remains unclear whether VOMS will be feasible or widely utilized among people who live in rural/remote geographic areas or among individuals who do not have reliable telephone, cellular service or Wi-Fi access [30]. Anxiety about confidentiality and fear of arrest likely poses barriers to using VOMS and potentially other harm reduction services.

## Future directions

Increasing awareness around harm reduction services and VOMS is still needed and awareness campaigns may be most effective via existing peer networks and grassroots outreach approaches. Further exploration of barriers, such as access to phone and data reception and emergency response times, for accessing VOMS, particularly in rural, remote and indigenous communities, is warranted to inform health promotion efforts. Prospective and epidemiological studies may help determine whether VOMS can make a population-level impact on health outcomes. Provision of mobile technologies (e.g., smartphones, Wi-Fi) to PWUS may be considered in future research to potentially improve access/utilization of VOMS among people who have unreliable or limited access to technology [28]. Decriminalizing the personal possession of substances nationally may help improve access to VOMS and other harm reduction interventions. More promotion/awareness about VOMS, in addition to a better understanding of the *Good Samaritan Drug Overdose Act*, increased research into EMS response times and the frequency of police involvement, are needed to improve uptake and understand VOMS's limitations better.

## Supplementary Information

**Additional file 1.** Interviewer Telephone Script.
