# Supplementary material for: Beliefs, attitudes and experiences of virtual overdose monitoring services from the perspectives of people who use substances in Canada: a qualitative study
Source: Harm Reduct J. 2023 Jun 24;20:80. doi: 10.1186/s12954-023-00807-9 (PMC10290798; doi:10.1186/s12954-023-00807-9)
Supplement: Supplementary file 1 — Additional file 1. Interviewer Telephone Script. [file 12954_2023_807_MOESM1_ESM.docx]

Interviewer Telephone script

| Hello, may I please speak to **<potential participant’s name>**? | |
| --- | --- |
| **Possible respondent responses** | **Suggested evaluator response** |
| *“These is no one here by that name”* | Sorry. Thank you for your time. **<Terminate call. Do not leave a message. Participation is confidential>** |
| *“They are not here/available”* | Is there another time I could reach them?  **<If yes, record date and time to call back>**  Ok, I’ll call back later. Thank-you for your time. **<Terminate call. Do not leave a message. Participation is confidential>** |
| *“They are too unwell to speak at the moment” (physically, emotionally, mentally unwell)* | Sorry to hear that. Is there another time I should try them?  **<If yes, record date and time to call back>**  Ok, I’ll call back later. Thank-you for your time. **<Terminate call. Do not leave a message. Participation is confidential>** |
| *“Yes, this is s/he speaking”* | My name is **<interviewer name>** and I am a researcher with Three Hive Consulting and we are conducting research with Dr. Monty Ghosh. **<insert name or recruitment agency/person>** indicated that you’d be interested in talking to us about virtual supervised consumption services.  Thank you for allowing **<insert name or recruitment agency/person>** to share your contact details with us and for your interest in the research study we are conducting.  I am calling today to ask if you wish to participate in the research study about virtual supervised consumption services. The study is being conducted by Dr. Monty Ghosh and is trying to learn if these services can be used to help other people who choose to use substances alone. We will talk to around 60 other people across Canada about these services. Would you be interested in hearing more about this study?  **<If ‘no’>** That is okay. Thank you for your time. Good bye. **<Terminate call>**  **<If ‘yes’, continue to information form and provide an overview of the information contained in the information form>** |

Virtual Supervised Consumption Services Research Study

# Information form

**Why do you want to talk to me?**

We would like to talk to you about something called virtual supervised consumption services. These services can help keep people safe when they use drugs and can be especially helpful for people who use alone. When individuals use some drugs, especially opioids, there can be a risk of fatal overdose.

Virtual supervised consumption services can be a telephone service or a smartphone app. Each of these methods monitor people who are using substances and activate emergency services or an emergency care plan if the person using becomes unresponsive.

These services include the National Overdose Response Service (NORS) phone hotline, the BRAVE app, and the DORS app. Anyone can call the number for NORS and someone will answer the hotline who uses substances, have used substances in the past, or have another personal experience with drug use. This service is confidential. That operator will ask for the caller’s location and what substances they are using. That operator will stay on the phone with the person using substances (the caller) and call for help if the caller becomes unresponsive. The operator will check in with the caller every 30 seconds to 1 minute. If the caller does not respond the co-created safety plan will be enacted (i.e., a close contact may be engaged to support the client in an overdose situation as provided during the phone call and emergency services are deployed). Callers will also have the option of accessing additional substance use-related and health service referrals from the line should they require it.

The BRAVE app is available to anyone in North America through the Google Play and Apple store and allows people who use alone to request remote supervision and anonymous overdose support. This virtual safe consumption space has person-to-person connection in which overdoses are detected and a community-led response is initiated. The person uses the digital app to request support before they use. A suitable supporter is identified through the app; the supporter is then connected to monitor the caller over the phone. The caller and supporter set agreements and establish trust and the supporter remains on the line to make sure the caller is safe. If the supporter suspects an overdose, they start the response process.

The DORS app is available to people in the greater Edmonton and Calgary area and can be downloaded from the Google Play and Apple store. The app allows people to confirm their location and start a timer when they are ready to use a substance. Once the timer counts down to 30 seconds an alarm is sounded that will increase in volume. The timer can be disabled or extended at any time by the app user. If the app user does not respond and the timer ends, an emergency alert is sent to a Response Centre. The Response Centre will try and call the app user to see if they require assistance. If the app user says they need assistance or they don’t answer, medical help is sent to their location. The DORS app also provides information on national and provincial addiction recovery supports and services.

Further details of these services can be found here:

NORS phone line: [https://www.nors.ca](https://www.nors.ca/)

BRAVE app: [https://www.thebraveapp.com](https://www.thebraveapp.com/)

DORS app: [https://www.dorsapp.ca](https://www.dorsapp.ca/)

We would like to learn if these services can be improved or modified to help other people who choose to use substances alone. We will talk to around 60 other people across Canada about these services.

**What are you asking me to do?**

We would like you to talk with us for 30 - 45 minutes on the telephone. Some things we would like to talk to you about are what you like and don’t like about virtual supervised consumption services, and also what you think should be changed to help other people who use or want to use alone.

You will talk to a trained evaluator from a contracted evaluation consulting firm called [Three Hive Consulting.](http://www.threehive.ca/) The conversation can be scheduled by clicking the Calendly link provided in the email. [Calendy](https://calendly.com/d/cgr-qr3-rgb/virtual-supervised-consumption-services-interview) is an automated scheduling platform that makes it easier for us to book a time with you. However, if you prefer you can call or email Stephanie Jones ([stephanie@threehive.ca](mailto:stephanie@threehive.ca) or 250.918.5351) to book a time. You do not have to share your real name if you don’t want to.

**Are there risks to me if I talk to you?**

Talking about some things in your life could bring out uncomfortable feelings. You do not have to answer any questions or talk about anything you do not want to talk about. It is up to you. You can change your mind about taking part later. All you have to do is tell us you want to stop.

While we will do everything we can to keep your identity and the information we talk about private, there is a very rare risk that if a privacy breach happened that certain people may be identified as using substances.

**Do you record the conversation?**

We would like to record what we talk about to make sure we get the right information; however, if you decide not to have what we talk about recorded that is okay. If you are okay with us recording what we talk about you should know it will be typed into a document and your (or any other) names will be removed. The recording will be erased from the recorder after the document is typed.

**Who will see or hear the information you collect?**

We will do everything we can to keep your identity and the information we talk about private. Only specific people from the research team and contracted evaluation firm (Three Hive) will have access to the name and other personal information provided, but no identifiable information will be included in any reports. As previously mentioned, you do not have to share your real name if you don’t want to. We may use a quote you say in our reporting; however, we will only use quotes where you cannot be identified. Once the report is written we will throw away all of this information.

**How does me talking to you help?**

You may not benefit from it personally. However, we might find out different things talking to you that we would not have known by just talking to people who design and implement the services. The information you tell us could help other people who use alone and think the services might help.

**What do I get for talking to you?**

You will get a $50 gift card as a thank you for your time.

**Do I have to talk to you?**

You do not have to talk to us. It’s up to you. If you want to talk to us, you can change your mind at any time. All you have to do is tell us.

**Who do I call if I have more questions?**

Dr. S. Monty Ghosh, Research Lead (403-367-5000)

Kristy Madsen, Three Hive Consulting Evaluation Lead (780-399-1273)

Virtual Supervised Consumption Services Research Study

# Statement and Signature for Verbal Consent

<Insert title of person who goes over information form> **Statement and Signature**

The undersigned <Insert title of person who goes over information form> hereby certifies that he/she has discussed the study with participant <insert client #> and has explained the information contained in the participant information form, including the reason for the conversation, the risks, and the potential benefits. The undersigned <Insert title of person who goes over information form> further certifies that the participant was encouraged to ask questions and that all questions were answered prior to obtaining verbal consent.

Signature _____________________  Date __________________________

Virtual Supervised Consumption Services Research Study

# **People Who Use Substances** Conversation Script and guide

**Introduction**

Thank you for agreeing to talk with me today. Some things we would like to talk to you about are what you like and don’t like about virtual supervised consumption services, and also what you think should be changed to help other people who use or want to use alone.

Virtual supervised consumption services include services such as the National Overdose Response Service (NORS) phone hotline, the BRAVE app, and the DORS app. Further details on these services can be found here:

NORS phone line: [https://www.nors.ca](https://www.nors.ca/)

BRAVE app: [https://www.thebraveapp.com](https://www.thebraveapp.com/)

DORS app: [https://www.dorsapp.ca](https://www.dorsapp.ca/)

There are no right or wrong answers to my questions. We are hoping the conversation won’t take more than 45 minutes.

Please know that I do not have a personal interest in virtual supervised consumption services in general, so please feel free to speak openly and honestly. Everything you say is voluntary and will be kept confidential to the extent explained earlier.

As a thank you for your time, you will receive a $50 visa gift card. At the end of the interview, I will just double check we have the correct information to get the card to you. **This will not be linked with your responses.**

Do you have any questions? Is it okay to proceed?

**<If yes,** proceed to age screening question>

<**If no,** ask if and what information needs to be clarified. Clarify and again ask if it is okay to proceed>

**Age Verification**

Before we begin, are you currently 18 years of age or older?

**<If yes,** proceed to first question>

<**If no,** thank the person for their time and let them know that the person must be 18 years of age to participate>

(The blue font are the HC Excellence sub-evaluation questions; all should be asked)

| **INTERVIEW GUIDE** | |
| --- | --- |
| **QUESTION** | **PROBE** |
| **Awareness** |  |
| How did you first hear of virtual supervised consumption services? | Friend?  Family?  Community agency?  Written material? E.g., naloxone kits, handouts from community agency, etc. |
| Do you recall what your understanding of these services were PRIOR to using them? | Did you know…?   - Who the VSC services were meant for? (i.e., people who use substances alone) - When the VSC services should be used? (i.e., before a person uses) |
| What is your understanding of the VSC services AFTER using them? | Anything that surprised you?  Anything changed? |
| Do you feel like you have enough information about these virtual supervised consumption services? | Why or why not?  What other information do you wish you had? |
| What information should other people who use substances and are thinking about using these services know? | Who the VSC services are meant for? (i.e., people who use substances alone)  When the VSC services should be used? (i.e., before a person uses) |
| If information about these services were included in naloxone kits to improve awareness, what key information should be included? | Anything that shouldn’t be included? I.e., that would turn someone off or make them not want to use the service?  Do you think putting information about VSC services in naloxone kits is a good way to spread awareness about the services? |
| Which information are you more likely to read: 1) information about these services on a small pamphlet inserted in naloxone kits, 2) information that is accessed through a QR code in the naloxone kit, or 3) a combination of both? | How much information is too much information? |
| Any other ideas on how to promote awareness of virtual supervised consumption services? How can we make more people aware of the service (best strategies)? | Anything specific to naloxone kits? |
| **Adoption** |  |
| Which type of virtual supervised consumption services have you used? | NORS telephone line  VSC app – which one?  Both?  Other? |
| Tell me why you decided to try <insert type of virtual supervised consumption service>? | Did COVID-19 factor into your decision at all? How? Did COVID-19 impact how acceptable it is to use VSCS?  What other factors played into your decision?   - Difficulty accessing safe consumption sites? - Safety at safe consumption sites? - Privacy and anonymity of VSCS? - Discrimination? |
| Do you feel VSC was timely given the COVID-19 pandemic? |  |
| Do you use virtual supervised consumption services every time you use? Why or why not? | Do you also use safe consumption sites? Why or why not? |
| What were some things you were worried about before trying <insert type of virtual supervised consumption service>? | Privacy and anonymity?  Being treated with dignity and respect?  Personal/cultural beliefs?  Has this changed now that you have used the service? Or do you still have any concerns? |
| Who do you think would benefit most from VSCS? | What setting would they be in? E.g., private accommodation etc. |
| What would impact people using the service? What do you think other people might be worried about? | What do you think are the barriers to people using the VSC services? (e.g., worried about sharing their information)  How do you think someone’s gender and cultural background may impact their acceptance of using VSCS in phone-based and app-based form?  For what reasons would someone use the service and not use the service?  Possession of a phone/cell phone?  Cultural or belief factors? |
| **Usability** |  |
| Do you find the service easy to use? | Do you prefer to use the phone line or the app? Why?  What aspects are easiest for each of the services? (*modify depending on which service they use*) |
| What are the difficult aspects of the phone-based or app-based service? (*modify based on which service they use*) |  |
| What would make the service easier to use in your setting? | For both phone and/or app-based (*depending on which service they use*)  Is there anything that would make you feel more comfortable using the service? |
| What, if any, recommendations do you have for how to improve virtual supervised consumptions services for people who use substances? | Anything it should *start* doing?  Anything it should *stop* doing?  In what ways can we improve upon the safety of both the phone and app-based service in your community? |
| **Acceptability** |  |
| What has been your experience when you’ve used <insert type of virtual supervised consumption service>? |  |
| When you think about your experiences with virtual supervised consumption services…. |  |
| …. did you feel like you were treated with dignity and respect? | Were your wants and needs respected? (i.e., care plan wishes?)  Why or why not? |
| …. did the operator help to alleviate your fears and reassure you? | Why or why not?  What would make you feel more comfortable with the service? |
| …. did the operator connect you with resources in the community? | Were the resources appropriate/sufficient? Why or why not? |
| What aspects of the service did you find particularly useful? What aspects did you find not useful? |  |
| **Appropriateness** | |
| Have you ever used virtual supervised consumption services for supports not related to opioid use? Was this primarily through the app or the phone?  Did the use of VSCS connect you with other resources in the community? | Mental health support?  Addiction support?  **If yes,** why did you choose to use this service?  **If yes,** did you find the support helpful? Why or why not?  What types of processes would need to be developed if VSCS were used for supports not related to substance use or to be used in your community? |
| In what ways, if at all, have virtual supervised consumption services helped you to safely use alone? | Do you feel the length of time spent with the operators are adequate?  Do you feel safe with the potential response times for an overdose through VSCS? |
| Are there opportunities to access virtual supervised consumption services in other settings? | Hospitals?  Community-based settings? Where else?  What barriers would there be?  How would this help you? |
| If virtual supervised consumption services didn’t exist, how would that impact you? | Your family/friends?  Your community?  The health system? |
| What about on the flip-side, are there any disadvantages to virtual supervised consumption services? | For people who use substances?  For community?  For health systems? |
| What, if any, recommendations do you have for how to improve virtual supervised consumptions services for others and your community? | People who are thinking about using?  Health care providers?  Others? |
| Is there anything else that I haven’t asked you about that you’d like to share? |  |
| **Snowball:** We are also looking to speak with other people who use substances and chat about their thoughts on Virtual Supervised Consumptions Services. We would also like to speak with family members as well. Can you think of anyone that might be interested in speaking with us? Please feel free to share my email and phone number with them and to reach out to me if they would like to schedule a time. They will also receive a gift card as a thank you for their time. | <interviewer to provide their email and phone number if participants show interest> |
| Only a few more quick questions. The following questions will be used to help us understand if virtual supervised consumption services affect different people and groups in different ways. Please note no information that identifies you, including the following information, will be reported with any of the other information you provided in the report. Again, you do not have to answers any of the questions if you do not want to. | |

| Demographic Questions | |
| --- | --- |
| Questions | **Probe** |
| 1. What is your age? |  |
| 1. Which gender do you most identify? | <Let person state gender. If a probe is required then….>  Agender  Genderfluid  Man  Non-binary  Trans Person  Two Spirit  Woman  Other: ____________  Prefer not to say |
| 1. Do you identify as a BIPOC person? | Do you identify as Indigenous? |
| 1. In which province or territory are you currently residing? |  |
| 1. Do you consider the place you currently live to be urban or rural? |  |

**As I mentioned, you will be receiving a $50 visa gift card as a thank you for participating in the interview. We can share the gift card electronically via email, or mail to your address. Which is easiest? Please could you share your details? This will not be linked to your responses in any way.**

Thank you so much for your time. Please feel free to contact me if you think of anything else that you’d wish to include. Take care.

## Appendix A: Support Services

Should the participant feel discomfort or triggered by the content in the conversation, please provide the following resources for them reach out to:

- For URGENT medical concerns, including overdose, please dial 911.
- To receive URGENT crisis support in Calgary, please call the Calgary Distress Centre at 403-266-HELP (4357).
- Outside of Calgary, please call 211 for assistance or Crisis Services Canada at 1-833-456-4566 (1-866-277-3553 in Quebec).
- To be connected with addictions services in Alberta, please call Health Link Alberta at 811.

## Appendix B: If the participant uses substances during the interview

- Tell the participant that our interview protocol states we must take their address and will call 9-1-1 in the case of an overdose
- If the participant **agrees to sharing their address and calling 9-1-1**, take their address, and continue the interview. Monitor the participant for signs of overdose (not responding, slurred speech, not making sense, slow/erratic breathing, choking sounds, [video call only] blue tint to skin (for lighter skinned people) or grey/ashen tint (for darker skinned people)
  - Check-in with the participant, ask them if they are good to continue at multiple points throughout the interview. If the above symptoms arise and the participant does not respond to calling their name, call 9-1-1 and let the operator know the participant’s address and that they are unresponsive (do not use term ‘overdose’ for stigmatizing reasons)
- If the participant **does not agree to sharing their address OR you are not comfortable in the situation,** tell the participant that you must end the interview. Give the participant the phone number for NORS (888-668-6677). Let the participant know that they are welcome to reschedule the interview at another time

Virtual Supervised Consumption Services Evaluation

# **People Who Use Substances** but do not use VSCS Interview Information form

**Why do you want to talk to me?**

We would like to talk to you about something called virtual supervised consumption services. These services can help keep people safe when they use drugs and can be especially helpful for people who use alone. When individuals use some drugs, especially opioids, there can be a risk of fatal overdose.

Virtual supervised consumption services can be a telephone service or a smartphone app. Each of these methods monitor people who are using substances and activate emergency services if the person using becomes unresponsive.

These services include the National Overdose Response Service (NORS) phone hotline, the BRAVE app, and the DORS app. Anyone can call the number for NORS and someone will answer the hotline who uses substances, have used substances in the past, or have another personal experience with drug use. This service is confidential. That operator will ask for the caller’s location and what substances they are using. That operator will stay on the phone with the person using substances (the caller) and call for help if the caller becomes unresponsive. The operator will check in with the caller every 30 seconds to 1 minute. If the caller does not respond the co-created safety plan will be enacted (i.e., a close contact may be engaged to support the client in an overdose situation as provided during the phone call and emergency services are deployed). Callers will also have the option of accessing additional substance use-related and health service referrals from the line should they require it.

The BRAVE app is available to anyone in North America through the Google Play and Apple store and allows people who use alone to request remote supervision and anonymous overdose support. This virtual safe consumption space has person-to-person connection in which overdoses are detected and a community-led response is initiated. The person uses the digital app to request support before they use. A suitable supporter is identified through the app; the supporter is then connected to monitor the caller over the phone. The caller and supporter set agreements and establish trust and the supporter remains on the line to make sure the caller is safe. If the supporter suspects an overdose, they start the response process.

The DORS app is available to people in the greater Edmonton and Calgary area and can be downloaded from the Google Play and Apple store. The app allows people to confirm their location and start a timer when they are ready to use a substance. Once the timer counts down to 30 seconds an alarm is sounded that will increase in volume. The timer can be disabled or extended at any time by the app user. If the app user does not respond and the timer ends, an emergency alert is sent to a Response Centre. The Response Centre will try and call the app user to see if they require assistance. If the app user says they need assistance or they don’t answer, medical help is sent to their location. The DORS app also provides information on national and provincial addiction recovery supports and services.

Further details on these services can be found here:

NORS phone line: [https://www.nors.ca](https://www.nors.ca/)

BRAVE app: [https://www.thebraveapp.com](https://www.thebraveapp.com/)

DORS app: [https://www.dorsapp.ca](https://www.dorsapp.ca/)

We would like to hear from the people these services could maybe be used by to learn if they can be used to help other people who choose to use substances alone. We will talk to around 60 other people across Canada about these services.

**What are you asking me to do?**

We would like you to talk with us for 30 - 45 minutes on the telephone. Some things we would like to talk to you about are what you think about virtual supervised consumption services, what you think you would like and not like about them, and also, what you think should be changed to help other people who use or want to use alone.

You will talk to a trained evaluator from a contracted evaluation consulting firm called [Three Hive Consulting.](http://www.threehive.ca/) The conversation can be scheduled by clicking the Calendly link provided in the email. [Calendy](https://calendly.com/d/cgr-qr3-rgb/virtual-supervised-consumption-services-interview) is an automated scheduling platform that makes it easier for us to book a time with you. However, if you prefer you can call or email Stephanie Jones ([stephanie@threehive.ca](mailto:stephanie@threehive.ca) or 250.918.5351) to book a time.

**Are there risks to me if I talk to you?**

Talking about some things in your life could bring out uncomfortable feelings. You do not have to answer any questions or talk about anything you do not want to talk about. It is up to you. You can change your mind about taking part later. All you have to do is tell us you want to stop.

While we will do everything we can to keep your identity and the information we talk about private, there is a very rare risk that if a privacy breach happened that certain people may be identified as using substances.

**Do you record the conversation?**

We would like to record what we talk about to make sure we get the right information; however, if you decide not to have what we talk about recorded that is okay. If you are okay with us recording what we talk about you should know it will be typed into a document and your (or any other) names will be removed. The recording will be erased from the recorder after the document is typed.

**Who will see or hear the information you collect?**

We will do everything we can to keep your identity and the information we talk about private. Only specific people from the research team and contracted evaluation firm (Three Hive) will have access to the name and other personal information provided, but no identifiable information will be included in any reports. As previously mentioned, you do not have to share your real name if you don’t want to. We may use a quote you say in our reporting; however, we will only use quotes where you cannot be identified. Once the report is written we will throw away all of this information.

**How does me talking to you help?**

You may not benefit from it personally. However, we might find out different things talking to you that we would not have known by just talking to people who design and implement the services. The information you tell us could help other people who use alone and think the services might help.

**What do I get for talking to you?**

You will get a $50 gift card as a thank you for your time.

**Do I have to talk to you?**

You do not have to talk to us. It’s up to you. If you want to talk to us, you can change your mind at any time. All you have to do is tell us.

**Who do I call if I have more questions?**

Dr. S. Monty Ghosh, Research Lead (403-367-5000)

Kristy Madsen, Three Hive Consulting Evaluation Lead (780-399-1273)

Virtual Supervised Consumption Services Evaluation

# Statement and Signature for Verbal Consent

<Insert title of person who goes over information form> **Statement and Signature**

The undersigned <Insert title of person who goes over information form> hereby certifies that he/she has discussed the evaluation with participant <insert client #> and has explained the information contained in the participant information form, including the reason for the interview, the risks, and the potential benefits. The undersigned <Insert title of person who goes over information form> further certifies that the participant was encouraged to ask questions and that all questions were answered prior to obtaining verbal consent.

Signature _________________________________

Date _____________________________________

Virtual Supervised Consumption Services Evaluation

# **People Who Use Substances** but do not use VSCS Interview Script and guide

**Introduction**

Thank you for agreeing to talk with me today. We would like to talk to you about something called virtual supervised consumption services. These services can help keep people safe when they use drugs and can be especially helpful for people who use alone. When individuals use some drugs, especially opioids, there can be a risk of fatal overdose. Virtual supervised consumption services can be a telephone service or a smartphone app. Each of these methods monitor people who are using substances and activate emergency services if the person using becomes unresponsive.

We would like to hear from people that these apps could try to help to learn if they can be used to help people who choose to use substances alone. Some things we would like to talk to you about are what you think about the apps, what you like and don’t like about the idea of the apps, and also what you think should be changed to help other people who use or want to use alone. There are no right or wrong answers to my questions. We are hoping the conversation won’t take more than 45 minutes.

Please know that I do not have a personal interest in virtual supervised consumption services in general, so please feel free to speak openly and honestly. Everything you say is voluntary and will be kept confidential to the extent explained earlier.

As a thank you for your time, you will receive a $50 visa gift card. At the end of the interview, I will just double check we have the correct information to get the card to you. **This will not be linked with your responses.**

Do you have any questions? Is it okay to proceed?

**<If yes,** proceed to age screening question>

<**If no,** ask if and what information needs to be clarified. Clarify and again ask if it is okay to proceed>

**Age Verification**

Before we begin, are you currently 18 years of age or older?

**<If yes,** proceed to first question>

<**If no,** thank the person for their time and let them know that the person must be 18 years of age to participate>

(The blue font are the HC Excellence sub-evaluation questions; all should be asked)

| **INTERVIEW GUIDE** | |
| --- | --- |
| **QUESTION** | **PROBE** |
| **Awareness** |  |
| Had you heard of virtual supervised consumption services before us contacting you? | **If yes,** do you recall where or how you first heard of virtual consumption sites? e.g., friend, family, community agency, written material such as naloxone kits etc. |
| Do you recall ever seeing information of virtual supervised consumption services in naloxone kits? | **If yes,** do you recall what that naloxone sticker/insert said about virtual supervised consumption services?  Do you think putting information about VSC services in naloxone kits is a good way to spread awareness about the services? |
| Do you have any suggestions for information that should be included in naloxone kits to improve awareness of virtual supervised consumption services? | Any other ideas on how to promote awareness of virtual supervised consumption services? |
| Which information are you more likely to read: 1) information about these services on a small pamphlet inserted in naloxone kits, 2) information that is accessed through a QR code in the naloxone kit, or 3) a combination of both? | How much information is too much information? |
| What is your understanding of virtual supervised consumption services? | Do you know...?   - Who the services are meant for? (i.e., people who use substances alone) - When the services should be used? (i.e., before the person uses)   Where did you get this information from? |
| I am going to briefly explain three examples of virtual supervised consumption services and then ask you what you think about them. The first is a phone hotline known as the National Overdose Response Services (NORS). Anyone can call the number for NORS and someone will answer the hotline who uses substances, have used substances in the past, or have another personal experience with drug use. This service is confidential. That operator will ask for the caller’s location and what substances they are using. That operator will stay on the phone with the person using substances (the caller) and call for help if the caller becomes unresponsive. The operator will check in with the caller every 30 seconds to 1 minute. If the caller does not respond the co-created safety plan will be enacted (i.e., a close contact may be engaged to support the client in an overdose situation as provided during the phone call and emergency services are deployed). Callers will also have the option of accessing additional substance use-related and health service referrals from the line should they require it.  The BRAVE app is available to anyone in North America through the Google Play and Apple store and allows people who use alone to request remote supervision and anonymous overdose support. This virtual safe consumption space has person-to-person connection in which overdoses are detected and a community-led response is initiated. The person uses the digital app to request support before they use. A suitable supporter is identified through the app; the supporter is then connected to monitor the caller over the phone. The caller and supporter set agreements and establish trust and the supporter remains on the line to make sure the caller is safe. If the supporter suspects an overdose, they start the response process.  The DORS app is available to people in the greater Edmonton and Calgary area and can be downloaded from the Google Play and Apple store. The app allows people to confirm their location and start a timer when they are ready to use a substance. Once the timer counts down to 30 seconds an alarm is sounded that will increase in volume. The timer can be disabled or extended at any time by the app user. If the app user does not respond and the timer ends, an emergency alert is sent to a Response Centre. The Response Centre will try and call the app user to see if they require assistance. If the app user says they need assistance or they don’t answer, medical help is sent to their location. The DORS app also provides information on national and provincial addiction recovery supports and services. | |
| **Adoption** |  |
| Do you know of anyone who has used any type of VSCS before? | **If yes**, do you recall which ones? |
| Would you ever use any type of virtual supervised consumption services? | **If no,** why would you not be willing to try VSCS?  **If yes,** which one or ones would you be most likely to try:  NORS telephone line  VSCS app – which one?  Other? |
|  | Tell me why you would be willing to try <insert type of virtual supervised consumption service>?  Would COVID-19 factor into your decision at all? How?  What other factors play into your willingness to try?   - Difficulty accessing safe consumption sites? - Safety at safe consumption sites? - Privacy and anonymity of VSCS? - Discrimination? |
| What are some things you may be worried about before trying VSCS? | Privacy and anonymity?  Being treated with dignity and respect?  Personal/cultural beliefs? |
| Who do you think would most benefit from VSCS? | What setting would they be in? (e.g., private accommodation) |
| Are there any other barriers to people using VSC services? (e.g., worried about sharing their information)? | For what reasons would someone use the service and not use the service?  How do you think someone’s gender and cultural background may impact their acceptance of using VSCS in phone-based and app-based form?  Possession of a phone or cell phone?  Cultural or belief factors? |
| Do you use supervised consumption sites? Why or why not? | **If yes,** do you use supervised consumption sites every time you use? |
| **Acceptability** |  |
| What would make you feel more comfortable with using the VSC service? | What, if any, other information would you like to know about VSCS prior to making your decision about using them? |
| Do you feel like you have enough information about VSCS prior to making your decision about using the service? | Why or why not?  What other information do you wish you had? |
| **Appropriateness** | |
| Would you ever use virtual supervised consumption services for supports not related to opioid use? | **If yes,** why would you choose to use this service?  Mental health support?  Addiction support?  What types of processes would need to be developed if VSCS were used for supports not related to substance use or to be used in your community? |
| In what ways, if at all, do you think virtual supervised consumption services would help you to safely use alone? | Do you feel safe with the potential response times for an overdose? |
| Are there opportunities to access virtual supervised consumption services in other settings? | Hospitals?  Community-based settings? Where else?  What barriers would there be?  How would this help you? |
| If virtual supervised consumption services didn’t exist, how would that impact you? | Your family/friends?  Your community?  The health system? |
| What about on the flip-side, are there any disadvantages to virtual supervised consumption sites? | For people who use substances?  For community?  For health systems? |
| **Feasibility** | |
| What, if any, recommendations do you have for how to improve virtual supervised consumptions services for people who use substances? And for the safety of PWUS? | Anything it should *start* doing?  Anything it should *stop* doing?  In what ways can we improve upon the safety of both the phone and app-based service in your community? |
| What, if any, recommendations do you have for how to improve virtual supervised consumptions services for others and your community? | PWUS and are contemplating using?  Health care providers?  Others? |
|  |  |
| Is there anything else that I haven’t asked you about that you’d like to share? |  |
| Snowball: We are also looking to speak with other people who use substances and chat about their thoughts on Virtual Supervised Consumptions Services. We would also like to speak with family members as well. Can you think of anyone that might be interested in speaking with us? Please feel free to share my email and phone number with them and to reach out to me if they would like to schedule a time. They will also receive a gift card as a thank you for their time. | <interviewer to provide their email and phone number if participants show interest> |
| Only a few more quick questions. The following questions will be used to help us understand if virtual supervised consumption services affect different people and groups in different ways. Please note no information that identifies you, including the following information, will be reported with any of the other information you provided in the previous questions. Again, you do not have to answer any of the questions if you do not want to. | |

| Demographic Questions | |
| --- | --- |
| Questions | **Probe** |
| 1. What is your age? |  |
| 1. Which gender do you most identify? | <Let person state gender. If a probe is required then….>  Agender  Genderfluid  Man  Non-binary  Trans Person  Two Spirit  Woman  Other: ____________  Prefer not to say |
| 1. Do you identify as a BIPOC person? | Do you identify as Indigenous? |
| 1. In which province or territory are you currently residing? |  |
| 1. Do you consider the place you currently live to be urban or rural? |  |

**As I mentioned, you will be receiving a $50 visa gift card as a thank you for participating in the interview. We can share the gift card electronically via email, or mail to your address. Which is easiest? Please could you share your details? This will not be linked to your responses in any way.**

Thank you so much for your time. Please feel free to contact me if you think of anything else that you’d wish to include. Take care.

# Appendix A: Telephone script scenarios

| **TELEPHONE SCRIPT** | |
| --- | --- |
| May I please speak to **<individual name>**? | |
| **Possible respondent responses** | **Suggested evaluator response** |
| *“These is no one here by that name”* | Sorry. Thank you for your time. **<Terminate call>** |
| *“They are not here/available”* | Is there another time I could reach **<him/her>?**  **<If yes, record date and time to call back>**  Ok, I’ll call back later. Thank-you for your time. **<Terminate call>** |
| *“They are too unwell to speak” (physically, emotionally, mentally unwell)* | Sorry to hear that. I will call back at a later time. **<Terminate call>** |
| *“Who’s asking?”* | My name is **<evaluator name>** and I am an evaluator with Three Hive Consulting. You indicated that you’d be interested in talking about virtual supervised consumption services. |
| *“Yes, this is s/he speaking”* | My name is **<evaluator name>** and I am an evaluator with Three Hive Consulting. You indicated that you’d be interested in talking about virtual supervised consumption services. We are talking with people familiar with the services to get their feedback. Do you have about 30 -45 minutes to talk with me?  **<If ‘no’>** Is there a better time to call?  **<If ‘no’>**  That is okay. Thank you for your time. **<Terminate call>**  **<If ‘yes’, continue to Consent>** |

## Appendix B: Support Services

Should the participant feel discomfort or triggered by the content in the survey, please provide the following resources for them reach out to:

- For URGENT medical concerns, including overdose, please dial 911.
- To receive URGENT crisis support in Calgary, please call the Calgary Distress Centre at 403-266-HELP (4357).
- Outside of Calgary, please call 211 for assistance or Crisis Services Canada at 1-833-456-4566 (1-866-277-3553 in Quebec).
- To be connected with addictions services in Alberta, please call Health Link Alberta at 811.

## Appendix C: If the participant uses substances during the interview

- Tell the participant that our interview protocol states we must take their address and will call 9-1-1 in the case of an overdose
- If the participant **agrees to sharing their address and calling 9-1-1**, take their address, and continue the interview. Monitor the participant for signs of overdose (not responding, slurred speech, not making sense, slow/erratic breathing, choking sounds, [video call only] blue tint to skin (for lighter skinned people) or grey/ashen tint (for darker skinned people)
  - Check-in with the participant, ask them if they are good to continue at multiple points throughout the interview. If the above symptoms arise and the participant does not respond to calling their name, call 9-1-1 and let the operator know the participant’s address and that they are unresponsive (do not use term ‘overdose’ for stigmatizing reasons)
- If the participant **does not agree to sharing their address OR you are not comfortable in the situation,** tell the participant that you must end the interview. Give the participant the phone number for NORS (888-668-6677). Let the participant know that they are welcome to reschedule the interview at another time
